# The Therapeutic Effect of Shugan Xiehuo Formula in the Female Rat Model with Central Precocious Puberty

**DOI:** 10.1155/2020/5916168

**Published:** 2020-12-17

**Authors:** Weiping Yin, Songmei Li, Kun Zhang, Yin Xu, Dianfei Ma, Tingting Shi, Lei Xiong, Jie Xia

**Affiliations:** ^1^Department of Pediatrics, Nanjing University of Chinese Medicine, Nanjing 210023, Jiangsu Province, China; ^2^Department of Pediatrics, Yunnan University of Chinese Medicine, Kunming 650500, Yunnan Province, China; ^3^Department of Pharmacy, Yunnan Provincial Hospital of Traditional Chinese Medicine, Kunming 650021, Yunnan Province, China; ^4^Department of Internal Medicine, Yunnan University of Chinese Medicine, Kunming 650500, Yunnan Province, China

## Abstract

Central precocious puberty (CPP) severely affects children's physical and mental health and needs to be treated promptly and effectively. This article aimed to research the therapeutic effect of Shugan Xiehuo Formula (SXF) on CPP. A female CPP rat model was established and then treated with leuprolide and different doses of SXF. Sex organ volume and index were measured. Ovaries and uteri were visualized by hematoxylin-eosin staining. The concentrations of follicle-stimulating hormone (FSH), luteinizing hormone (LH), prolactin (PRL), and estradiol (E2) in peripheral blood were determined. The expression levels of gonadotropin-releasing hormone (GnRH), gonadotropin-releasing hormone receptor (GnRHR), estrogen receptor alpha (ER*α*), and G protein-coupled receptor 30 (GPR30) in the hypophysis were investigated by Real-Time Quantitative Reverse Transcription PCR and western blot. GnRH expression in the hypothalamus and GnRHR expression in the ovary were detected by immunohistochemistry. SXF reduced the volume of the bilateral ovaries, as well as the volumes of the uterus, hypothalamus, and hypophysis in the female CPP rats and diminished the index of the ovary, uterus, hypothalamus, and hypophysis in the female CPP rats (*P* < 0.05 or *P* < 0.01). SXF treatment inhibited follicle maturation and uterine wall thickening in the female CPP rats. SXF decreased the concentrations of FSH, LH, PRL, and E2 in the peripheral blood in the female CPP rats (*P* < 0.01 or *P* < 0.001). SXF suppressed the expressions of GnRH, GnRHR, ER*α*, and GPR30 in the hypophysis (*P* < 0.05), the expression of GnRH in the hypothalamus (*P* < 0.01), and the expression of GnRHR in the ovaries (*P* < 0.001) of the female CPP rats. Overall, our study revealed that SXF had therapeutic effects on CPP in female rats. This is worthy of promoting clinically.

## 1. Introduction

Central precocious puberty (CPP) is caused by premature activation of the hypothalamic-pituitary-gonadal (HPG) axis, which ultimately leads to the premature development of secondary sexual characteristics in children [1–3]. According to reports, 1 out of every 5,000 to 10,000 children develops CPP, and, in general, the incidence of CPP in girls is much higher than in boys [4]. If CPP is not properly treated, it affects a child's growth and leads to a shorter height in adulthood than normal adults. CPP results in a series of psychological and physical problems in children suffering from this disease, which is a concern for their parents and society.

In recent decades, the main treatment strategy of CPP has been the application of gonadotropin-releasing hormone analogs (GnRHa) [5, 6]. This treatment method has been shown to effectively inhibit the hypothalamic-pituitary-gonadal axis, thereby achieving the treatment aims [6]. Unfortunately, although GnRHa has been found to be conducive in the treatment of CPP, the benefits of GnRHa for growth in CPP children are still uncertain because of the lack of sufficient randomized controlled studies. In addition, GnRHa is expensive, placing a financial burden on the families of the patients. Recently, a common Chinese medicine formula, Shugan Xiehuo Formula (SXF), was found to have therapeutic effects on CCP in children. The researchers revealed that the total effective rate of SXF on female patients with CPP was as high as 88.89%. Moreover, SXF treatment significantly diminished the volume of the ovaries, as well as the sex hormone levels [7]. In the female CPP rat model, the intervention of SXF reduced gonadotropin-releasing hormone (GnRH) mRNA expression in the hypothalamus and gonadotropin-releasing hormone receptor (GnRHR) mRNA expression in the hypophysis. High expressions of GnRH and GnRHR are important indicators of the activation of the HPG axis. Thus, SXF was considered for treating CPP due to its suppressing of the activation of the HPG axis [8]. However, more evidence should be presented to support SXF in the clinical treatment of CPP.

In this research, the female rat model with CPP was established via subcutaneous injection of N-methyl-DL-aspartic acid (NMA). Different doses of SXF were used to treat CPP in the female rats so that the therapeutic effect of SXF on CPP could be explored. This study provides a reliable theoretical basis for the treatment of CPP with SXF.

## 2. Materials and Methods

### 2.1. Ingredients of SXF

Every 100 g of SXF was composed of the following ingredients: *Chinese thorowax root* (10 g), *Angelica sinensis* (10 g), *white peony root* (10 g), *Poria cocos Wolf* (10 g), *Atractylodis macrocephalae rhizoma* (10 g), *peppermint* (10 g), *Ecliptae herba* (10 g), *Ligustrum lucidum* (10 g), *Spica prunellae* (10 g), and *Radix glycyrrhizae preparata* (10 g). All Chinese medicine ingredients were purchased from Jiangyin Tianjiang Pharmaceutical Co., Ltd. (Jiangsu, China). Each Chinese medicine ingredient was a solid raw material and weighted to 10 g using an electronic scale. The ten Chinese medicine ingredients were mixed and diluted in distilled water for 30 min with boiling. Following filtration, the residue of the Chinese medicine was discarded. The liquid medicine (SXF) was placed in a 4°C refrigerator for storage.

### 2.2. Animals

A total of 60 female Sprague-Dawley rats (71.15 ± 8.22 g) was commercially provided by Shanghai Laboratory Animal Co. Ltd. (Shanghai, China). Rats were kept separately in cages in an SPF laboratory animal room at 22 ± 1°C and 55% ± 5% humidity with free access to water and food. The light/dark cycle was 12 h. All animal experiments in this study were approved by the Animal Ethics Committee (Grant no. R-082018033).

### 2.3. Construction and Treatment of the Female CPP Rat Model

Rats were randomly divided into six groups (ten rats per group), which were named as follows: control group, CPP group, CPP-LP group, CPP-HD-SXF group, CPP-MD-SXF group, and CPP-LD-SXF group. Rats of the control group were injected subcutaneously with 0.9% sodium chloride (0.2 mL/time) at 14 : 00 and 16 : 00 daily. To construct the CPP model, rats in the other five groups were subjected to daily subcutaneous injections of NMA (40 mg/kg; Solarbio, Beijing, China) at 14 : 00 and 16 : 00. Meanwhile, at 9 : 00 every day, rats in the control group and CPP group were given a gavage of 0.9% sodium chloride injection (1.0 mL per rat). Rats in the CPP-LP group were injected intramuscularly with leuprolide (LP) acetate microspheres (100 *μ*g/kg). At the same time point, rats of the CPP-LD-SXF group, CPP-MD-SXF group, and CPP-HD-SXF group were subjected to SXF gavages of 2.13 g/kg, 4.25 g/kg, and 8.50 g/kg, respectively.

The vaginal opening of the rats in each group was observed at 8 : 30 every day. When the vaginal opening of the rats in the CPP group was observed to be open, the injections of NMA were stopped. At the same time, the 0.9% sodium chloride injections in the rats of the control group were stopped. The injections of NMA for rats in the CPP-LP group, CPP-LD-SXF group, CPP-MD-SXF group, and CPP-HD-SXF group were also stopped. In the CPP group, rats with open vaginal openings were subjected to vaginal smears. The vaginal smears were observed under a microscope to determine the sexual cycle of the rats. The rats in the CPP group were sacrificed at the first preestrus stage. The rats in the other five groups were also sacrificed at the same time. It should be noted that before the rats were sacrificed, their body weights were measured and peripheral blood was obtained. After the rats had been sacrificed, the bilateral ovaries, uterus, hypophysis, and hypothalamus of each rat were obtained, weighed, and stored at −80°C.

### 2.4. Determination of Hormone Concentrations in Peripheral Blood

Peripheral blood from each rat was obtained and concentrated for 5 min at 3000 × g and 4°C to collect the serum. The concentrations of follicle-stimulating hormone (FSH), luteinizing hormone (LH), prolactin (PRL), and estradiol (E2) in the serum were determined by a radioimmunoassay testing kit (Hengyuan Biotechnology Co., Ltd., Shanghai, China).

### 2.5. Measurement of Sex Organ Index

Before the rats were sacrificed, the body weight of each rat was measured. After the rats were sacrificed, the weights of the ovaries, uterus, hypophysis, and hypothalamus were also measured. The ratio of ovary weight to body weight, uterus weight to body weight, hypophysis weight to body weight, and hypothalamus weight to body weight were subsequently calculated.

### 2.6. Hematoxylin-Eosin (HE) Staining

The ovaries and uteri of rats were fixed with 4% paraformaldehyde for 24 h at 4°C. Gradient alcohol was used to dehydrate. After treatment with xylene, the ovaries and uteri were embedded in paraffin and sectioned at 5 *μ*m. The sections were dried in an oven at 45°C. Thereafter, xylene was used to dewax the sections, and gradient alcohol was applied to hydrate the sections. The sections were subsequently stained with hematoxylin and eosin. After being dehydrated with alcohol and transparentized by xylene, sections were sealed in neutral resin and placed under a microscope to observe the growth of the follicles and the thickness of the uterine wall.

### 2.7. Quantitative Real-Time Polymerase Chain Reaction (qRT-PCR)

Sex hormone receptor-related gene expression in the hypophysis, including GnRH, GnRHR, estrogen receptor alpha (ER*α*), and G protein-coupled receptor 30 (GPR30), was investigated by qRT-PCR. In general, the hypophysis tissues were lysed on ice with the addition of TRIZOL reagent (Thermo Fisher Scientific, Waltham, MA, USA) to extract total RNA. PrimeScriptTMRT kit (TaKaRa, Shiga, Japan) was used for the reverse transcription reaction with 5 *μ*g of total RNA sample to synthesize cDNA template. Thereafter, a total of 2 *μ*L cDNA template was subjected to the PCR amplification reaction in a 20 *μ*L reaction system. The amplification reaction conditions were 40 cycles of 95°C for 30 s, 95°C for 15 s, and 62°C for 20 s. *β*-actin was used as the control. Primers were as follows: GnRH, forward: 5′-CCGCTGTTGTTCTGTTGACT-3′, reverse: 5′-GCAGATCCCTAAGAGGTGAA-3′. GnRHR, forward: 5′-TCACTCAGCCCTTAGCTGTCC-3′, reverse: 5′-GAAGGCTTCATGCCACCATTG-3′. ER*α*, forward: 5′-TCAGGCTACCATTACGGAGT-3′, reverse: 5′-CGCTTGTGCTTCAACATTCT-3′. GPR30, forward: 5′-TCATTTCTGCCATGCACCCA-3′, reverse: 5′-GTGGACAGGGTGTCTGATGT-3′. *β*-actin, forward: 5′-GGAGATTACTGCCCTGGCTCCTA-3′, reverse: 5′-GACTCATCGTACTCCTGCTTGCTG-3′. Finally, the 2^-ΔΔCt^ method was used for determining the relative mRNA expression.

### 2.8. Western Blot

Hypophysis tissues were ground into powder in liquid nitrogen. Cell lysate was added into the hypophysis tissue powder on ice to collect total protein. The total protein concentration was determined using the BCA assay kit (Pierce Chemical Company, Rockford, IL, USA). Sodium dodecyl sulphate polyacrylamide gel electrophoresis was used for the separation of proteins in each sample. After being transferred onto a polyvinylidene fluoride membrane, total protein was blocked with 5% skim milk for 1 h. Rabbit anti-GnRH, anti-GnRHR, anti-ER*α*, and anti-GPR30 primary antibodies (1 : 1000, Cell Signaling, Danvers, MA, USA) were incubated with the membrane overnight at 4°C. The membrane was then washed with Tris-buffered saline/0.1% Tween three times for 10 min per time. Thereafter, horseradish peroxidase-labeled goat anti-rabbit IgG secondary antibody (1 : 5000, Solarbio, Beijing, China) was added onto the membrane for 1 h incubation at room temperature. The membrane was washed three times with TBST. The protein bands were visualized using enhanced chemiluminescence reagent. *β*-Actin was used as the control.

### 2.9. Immunohistochemistry

The expressions of GnRH in the hypothalamus and GnRHR in the ovary were detected by immunohistochemistry. In short, the hypothalamus and ovary were fixed with 4% paraformaldehyde and then sectioned with a thickness of 5 *μ*m. The sections were blocked with 5% bovine serum albumin for 1 h. Rabbit anti-GnRH and anti-GnRHR (1 : 250, Lichen Biotechnology Co., Ltd., Shanghai, China) were used to treat the sections overnight at 4°C. Subsequently, sections were incubated for 2 h at room temperature with biotin-labeled anti-rabbit IgG antibody (1 : 200, Solarbio, Beijing, China). The sections were then stained with 3,3-diaminobenzidine and counterstained with hematoxylin. After being dehydrated and transparentized, the sections were sealed in neutral resin and observed under a microscope. Brown-yellow particles were considered to be positive GnRH and GnRHR expression signals.

### 2.10. Statistical Analysis

In this research, all data are expressed as the mean ± standard deviation based on three independent repeated trials. SPSS 19 software (SPSS Inc., Chicago, IL, USA) was used to process the data. This data included the ratios of ovary weight to body weight, uterus weight to body weight, hypothalamus weight to body weight, and hypophysis weight to body weight. Also included were the concentrations of FSH, LH, PRL, and E2 in the peripheral blood, and GnRH, GnRHR, ER*α*, and GPR30 protein expression in the hypophysis. GraphPad Prism (version 5, La Jolla, CA, USA) was used for generating the statistical graphs. Two-tailed paired Student's *t*-test was responsible for the comparisons between the two groups. One-way analysis of variance was applied for comparisons among at least three groups following Tukey's post hoc test. *P* < 0.05 indicated a statistically significant difference.

## 3. Results

### 3.1. SXF Reduced the Volume of the Sex Organs in the Female CPP Rat Model

**Figure 1:**
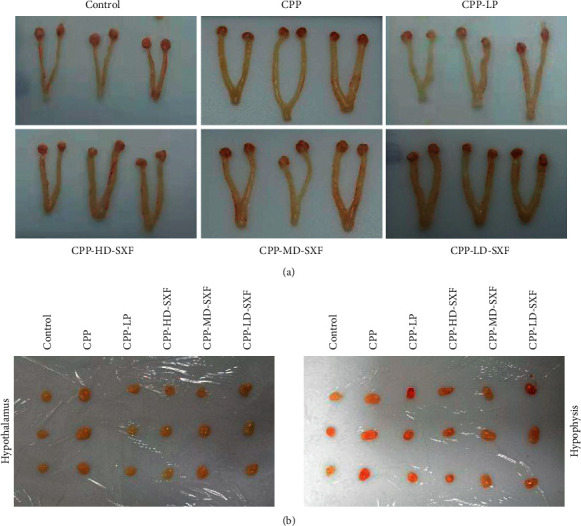
SXF reduced the volume of sex organs in female rat model with CPP. (a) The bilateral ovaries and uterus of female rat model with CPP was obtained and photographed after being sacrificed. (b) The hypothalamus and hypophysis of female rat model with CPP were obtained and photographed after being sacrificed.

After the rats were sacrificed, the bilateral ovaries, uterus, hypothalamus, and hypophysis of the rats in each group were obtained and photographed. As shown in Figures 1(a) and 1(b), the bilateral ovaries, uterus, hypothalamus, and hypophysis of rats in the CPP group were larger relative to the control group. Interestingly, compared with the CPP group, the volumes of the bilateral ovaries, uterus, hypothalamus, and hypophysis of rats in the CPP-LP group, CPP-HD-SXF group, CPP-MD-SXF group, and CPP-LD-SXF group were all reduced. Among the three groups treated with different SXF concentrations, rats in the CPP-HD-SXF group exhibited the smallest volume of bilateral ovaries, uterus, hypothalamus, and hypophysis.

### 3.2. SXF Reduced the Sex Organ Index in the Female CPP Rat Model

**Figure 2:**
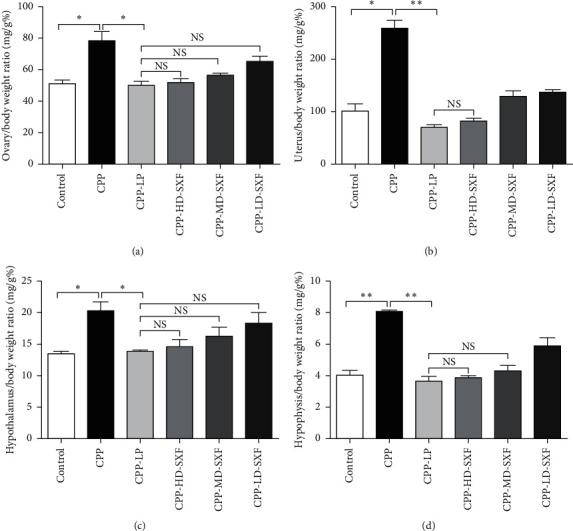
SXF reduced sex organ index in female rat model with CPP. (a) SXF decreased ovary index in female rat model with CPP. (b) SXF reduced uterus index in female rat model with CPP. (c) SXF declined hypothalamus index in female rat model with CPP. (d) SXF decreased hypophysis index in female rat model with CPP. ^*∗*^*P* < 0.05. ^*∗∗*^*P* < 0.05. NS indicates that there was no significant statistical difference between the two groups.

The sex organ indexes of the rats were monitored, including the ovary index, uterus index, hypothalamus index, and hypophysis index. The ovary index of rats in the CPP group was elevated compared with that of rats in the control group (*P* < 0.05). However, relative to the CPP group, rats of the CPP-LP group exhibited a lower ovary index (*P* < 0.05). Compared with the CPP-LP group, the ovary index of rats in the CPP-HD-SXF group, CPP-MD-SXF group, and CPP-LD-SXF group was not obviously changed (Figure 2(a)). Furthermore, a higher uterus index was found in the rats of the CPP group when compared with that of rats in the control group (*P* < 0.05). In comparison with the CPP group, the uterus index of the rats in the CPP-LP group was markedly reduced (*P* < 0.01). The uterus index difference between the CPP-LP group and CPP-HD-SXF group was not statistically significant (Figure 2(b)). In terms of the hypothalamus index, rats in the CPP group presented with a much higher hypothalamus index than those of the control group (*P* < 0.05). Conversely, the hypothalamus index of rats in the CPP-LP group was lower than that of rats in the CPP group (*P* < 0.05). No significant change in the hypothalamus index was observed in the CPP-HD-SXF group, CPP-MD-SXF group, and CPP-LD-SXF group when compared with the CPP-LP group (Figure 2(c)). Moreover, the hypophysis index of the rats in the CPP group was significantly increased relative to that of rats in the control group (*P* < 0.01). On the contrary, rats in the CPP-LP group exhibited a remarkably decreased hypophysis index when compared with those in the CPP group (*P* < 0.01). Compared with the CPP group, the hypophysis index in the rats of the CPP-HD-SXF group and CPP-MD-SXF group was not changed (Figure 2(d)).

### 3.3. SXF Inhibited the Maturation of Follicles and Thickening of the Uterine Wall in the Female CPP Rat Model

**Figure 3:**
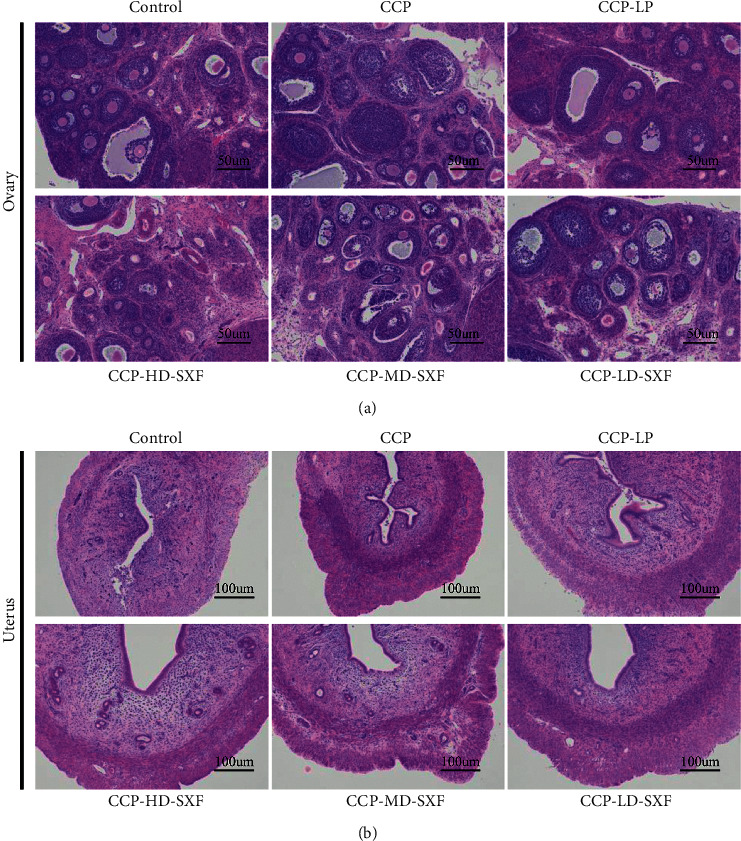
SXF inhibited maturation of follicles and thickening of the uterine wall in female rat model with CPP. (a) SXF inhibited maturation of follicles in female rat model with CPP. (b) SXF inhibited thickening of the uterine wall in female rat model with CPP.

The ovaries and uteri of the rats were stained with HE to observe follicle growth and the thickness of the uterine wall. For rats in the control group, the follicles were mainly primordial follicles, primary follicles, and a small number of secondary follicles. Tertiary follicles were not found in the ovaries of rats in the control group. However, multiple secondary follicles and tertiary follicles were observed in the ovaries of rats in the CPP group. Relative to the CPP group, the number of secondary follicles and tertiary follicles were decreased in the CPP-LP group, CPP-HD-SXF group, CPP-MD-SXF group, and CPP-LD-SXF group. The CPP-HD-SXF group exhibited fewer secondary follicles and tertiary follicles than those found in the CPP-MD-SXF group and CPP-LD-SXF group (Figure 3(a)). In addition, compared with the control group, the uterine wall of rats in the CPP group was obviously thickened. Compared with the CPP group, the uterine wall thickness of the rats in the CPP-LP group, CPP-HD-SXF group, CPP-MD-SXF group, and CPP-LD-SXF group was diminished. The uterine wall thickness of the rats in the CPP-HD-SXF group was much thinner than that in the CPP-MD-SXF group and CPP-LD-SXF group (Figure 3(b)).

### 3.4. SXF Decreased FSH, LH, PRL, and E2 Concentrations in the Peripheral Blood in the Female CPP Rat Model

**Figure 4:**
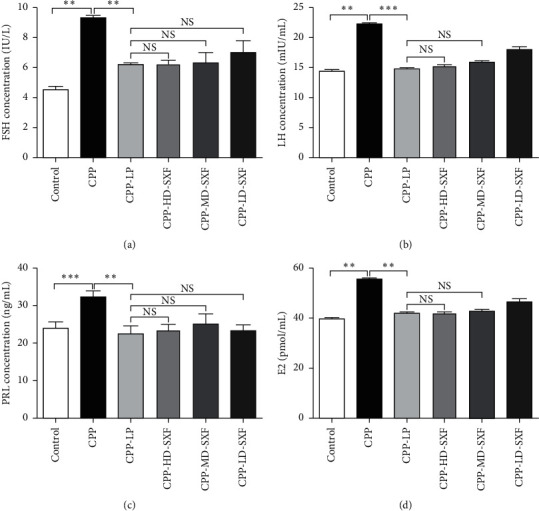
SXF declined FSH, LH, PRL, and E2 concentration in peripheral blood in female rat model with CPP. (a) SXF decreased FSH concentration in the peripheral blood of female rat model with CPP. (b) SXF reduced LH concentration in the peripheral blood of female rat model with CPP. (c) SXF declined PRL concentration in the peripheral blood of female rat model with CPP. (d) SXF diminished E2 concentration in the peripheral blood of female rat model with CPP. ^*∗*^*P* < 0.05. ^*∗∗*^*P* < 0.05. NS indicates that there was no significant statistical difference between the two groups.

The concentrations of FSH, LH, PRL, and E2 in the peripheral blood of the rats were detected. Rats in the CPP group showed increased FSH concentration compared with those in the control group (*P* < 0.01). The FSH concentration was significantly decreased in rats in the CPP-LP group when compared with those in the CPP group (*P* < 0.01). Relative to the CPP-LP group, the FSH concentrations in rats of the CPP-HD-SXF group, CPP-MD-SXF group, and CPP-LD-SXF group were not obviously changed (Figure 4(a)). In addition, the LH concentration of the rats in the CPP group was distinctly higher than those in the control group (*P* < 0.01). However, relative to the LH concentration of rats in the CPP group, it was reduced in the CPP-LP group (*P* < 0.001). Compared with the CPP-LP group, a change in the LH concentration was not obvious in the CPP-HD-SXF group and CPP-MD-SXF group (Figure 4(b)). Detection of the PRL concentration showed that, compared with the control group, a remarkably higher PRL concentration was found in the rats in the CPP group (*P* < 0.001). Compared with the CPP group, the PRL concentration was dramatically diminished in rats in the CPP-LP group (*P* < 0.01). Relative to the CPP-LP group, obvious changes in PRL concentration were not observed in the rats in the CPP-HD-SXF group, CPP-MD-SXF group, and CPP-LD-SXF group (Figure 4(c)). Moreover, the E2 concentration was much higher in rats in the CPP group than those in the control group (*P* < 0.01). In contrast, a prominently lower E2 concentration was observed in rats in the CPP-LP group when compared with those in the CPP group (*P* < 0.01). No significant statistical difference in E2 concentration was observed in the CPP-HD-SXF group and CPP-MD-SXF group relative to the CPP-LP group (Figure 4(d)).

### 3.5. SXF Suppressed the Expression of Sex Hormone Receptor-Related Genes in the Female CPP Rat Model

**Figure 5:**
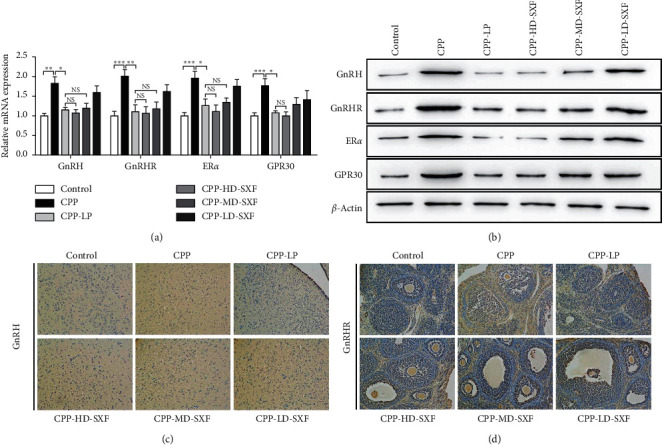
SXF suppressed the expression of sex hormone receptor-related genes in female rat model with CPP. (a) SXF suppressed GnRH, GnRHR, ER*α*, and GPR30 mRNA expression in hypophysis in female rat model with CPP. (b) SXF reduced GnRH, GnRHR, ER*α,* and GPR30 proteins expression in hypophysis in female rat model with CPP. (c) SXF reduced the positive signals of GnRH protein in hypothalamus according to immunohistochemistry. (d) Immunohistochemistry indicated that SXF decreased the positive signals of GnRHR protein in ovary. ^*∗*^*P* < 0.05. ^*∗∗*^*P* < 0.05. ^*∗∗∗*^*P* < 0.05. NS indicates that there was no significant statistical difference between the two groups.

The expression of the sex hormone receptor-related genes in the hypophysis was explored. qRT-PCR results indicated that rats in the CPP group had distinctly higher mRNA expression of GnRH, GnRHR, ER*α*, and GPR30 than those in the control group (*P* < 0.01 or *P* < 0.001). Relative to the CPP group, the mRNA expressions of GnRH, GnRHR, ER*α*, and GPR30 were markedly reduced in rats in the CPP-LP group (*P* < 0.05 or *P* < 0.01). Compared with the CPP-LP group, changes in GnRH, GnRHR, and ER*α* mRNA expression were not obvious in the CPP-HD-SXF group and CPP-MD-SXF group. Meanwhile, no statistically significant difference was found in GPR30 mRNA expression between the CPP-LP group and CPP-HD-SXF group (Figure 5(a)). According to western blot, obviously increased GnRH, GnRHR, ER*α*, and GPR30 protein expressions were found in rats in the CPP group when compared with those in the control group. However, relative to the CPP group, the protein expressions of GnRH, GnRHR, ER*α*, and GPR30 were all reduced in the CPP-LP group. When compared to the CPP-LP group, no changes were found in GnRH, GnRHR, and GPR30 protein expression in rats in the CPP-HD-SXF group and CPP-MD-SXF group. At the same time, ER*α* expression was not changed in rats in the CPP-HD-SXF group when compared with those in the CPP-LP group (Figure 5(b)).

Furthermore, GnRH expression in the hypothalamus and GnRHR expression in the ovaries were explored by immunohistochemistry. As shown in Figure 5(c) and 5(d), rats of the CPP group exhibited more positive signals of GnRH and GnRHR proteins than the control group. Conversely, when compared with the CPP group, the positive signals of GnRH and GnRHR proteins were significantly reduced in rats in the CPP-LP group. No changes occurred in the positive signals of GnRH and GnRHR proteins in rats in the CPP-HD-SXF group, CPP-MD-SXF group, and CPP-LD-SXF group relative to those in the CPP-LP group.

## 4. Discussion

It is well known that the onset of puberty begins with the increase of GnRH released by the hypothalamus, which in turn leads to the activation of the HPG axis [9]. CPP is caused by early activation of the HPG axis, which results in the early development of the uterus, ovary, and early appearance of the corpus luteum. This process eventually causes the occurrence of CPP [2, 10]. CPP affects the physical development and mental health of children but also increases the risk of metabolic diseases related to CPP [11]. Therefore, the implementation of effective CPP treatment is crucial. This article used the female CPP rat model to investigate the therapeutic effect of SXF for the treatment of CPP.

The function of NMA to promote precocious puberty in female rats has been confirmed in previous studies, and NMA at a dose of 40 mg/kg has been found to be effective in establishing the female rat model with precocious puberty [12, 13]. Thus, in this research, the female rat model with CPP was successfully established by subcutaneous injections of NMA (40 mg/kg). LP is a type of GnRH analog, which is commonly used in the clinical treatment of childhood CPP [14, 15]. Research has shown that LP at a dose of 100 *μ*g/kg can control the development of gonads in rats [16]. In this study, female CPP rats treated with LP were served as the positive control. We noticed that, similar to LP, SXF possessed an effective therapeutic effect in female rats with CPP. This effect included the inhibition of both follicular development and thickening of the uterine wall, a reduction in sex organ volume and index, and the suppression of hormone levels and expression of sex hormone receptor-related genes. This evidence clearly revealed the therapeutic effect of SXF on CPP.

In the female CPP rat model, an increased expression of GnRH was observed in the hypothalamus and hypophysis. At the same time, elevated expression of GnRHR in the hypophysis and ovaries was also detected. GnRH is the key to regulating reproductive function [17]. The concentration of GnRH is difficult to detect in the blood, and thus the detection of GnRH expression in the hypothalamus is commonly used [10]. Researchers have reported that elevated secretion of GnRH in the hypothalamus can combine with GnRHR in the hypophysis, thereby promoting the secretion of LH and FSH. Thereafter, LH and FSH further stimulate the synthesis of E2 and PRL by acting on the gonads, which ultimately promotes the development of the sex organs, such as the development of the uterus and ovaries, as well as the formation and maturation of the follicles [18]. Thus, LH and FSH levels in the blood are important indicators for the clinical evaluation of the therapeutic effect of CPP. The results from this paper indicated that SXF effectively reduced the expression of GnRH and GnRHR, as well as the levels of FSH, LH, PRL, and E2 in the peripheral blood in the female CPP rat model. As a result, the sex organ volume and index, the thickening of the uterine wall, and the maturation of the follicles were subsequently suppressed. Therefore, SXF might achieve therapeutic effects on CPP by inhibiting GnRH expression in the hypothalamus and GnRHR expression in the hypophysis. This further suppressed the secretion of estrogen, thereby inhibiting the development of the sex organs and delaying the activation of the HPG axis.

This study also monitored that in the female CPP rat model, the expressions of ER*α* and GPR30 in the hypophysis were aberrantly elevated. Interestingly, treatment with SXF prominently reduced the expression levels of ER*α* and GPR30 in the hypophysis in the female CPP rat model. Estrogen is a crucial factor that triggers CPP and the onset of puberty. ER*α* and GPR30 are two kinds of estrogen receptors that can mediate the expression of estrogen. The increased expressions of ER*α* and GPR30 can enhance the sensitivity of the estrogen receptor, which is closely related to the etiology of CPP [19–21]. It has also been reported that GPR30 can modulate the homeostasis of ER*α* [22]. Data from this research illustrated that SXF could suppress the expression of ER*α* and GPR30 in the hypophysis in the female CPP rat model.

Of course, this study has limitations. SXF contains ten Chinese medicine products. In this study, we were unable to research the impact of each product on CPP. This will be the focus of our future research.

## 5. Conclusions

Collectively, this article discovered that SXF has effective therapeutic effects on the female CPP rat model. SXF reduced the levels of FSH, LH, PRL, and E2 but also suppressed the expression of sex hormone receptor-related genes such as GnRH, GnRHR, ER*α*, and GPR30. This evidence reveals the therapeutic effect of SXF on CPP. We will conduct more research in the future to provide a deeper theoretical basis for SXF in the treatment of CPP.

## Data Availability

The datasets analyzed during the current study are available from the corresponding author on reasonable request.

## Abbreviations

CPP: Central precocious puberty
SXF: Shugan Xiehuo formula
HD: High dose
MD: Medium dose
LD: Low dose
FSH: Follicle-stimulating hormone
LH: Luteinizing hormone
PRL: Prolactin
E2: Estradiol
GnRH: Gonadotropin-releasing hormone
GnRHR: Gonadotropin-releasing hormone receptor
ER*α*: Estrogen receptor alpha
GPR30: G protein-coupled receptor 30
HPG: Hypothalamic-pituitary-gonad
GnRHa: Gonadotropin-releasing hormone analogs
NMA: N-methyl-DL-aspartic acid
HE: Hematoxylin-eosin
qRT-PCR: Quantitative real-time polymerase chain reaction
SDS-PAGE: Sodium dodecyl sulphate polyacrylamide gel electrophoresis
PVDF: Polyvinylidene fluoride
TBST: Tris-buffered saline/0.1% tween
ECL: Enhanced chemiluminescence
DAB: 3, 3-Diaminobenzidine.
